# A fully automated and reproducible level-set segmentation approach for generation of MR-based attenuation correction map of PET images in the brain employing single STE-MR imaging modality

**DOI:** 10.1186/2197-7364-1-S1-A48

**Published:** 2014-07-29

**Authors:** Anahita Fathi Kazerooni, Mohammad Hadi Aarabi, Mohammadreza Ay, Hamidreza Saligheh Rad

**Affiliations:** Quantitative MR Imaging and Spectroscopy Group, Research Center for Cellular and Molecular Imaging, Tehran University of Medical Sciences, Tehran, Iran; Department of Medical Physics and Biomedical Engineering, Tehran University of Medical Sciences, Tehran, Iran; Medical Imaging Systems Group, Research Center for Cellular and Molecular Imaging, Tehran University of Medical Sciences, Tehran, Iran

Generating MR-based attenuation correction map (*μ*-map) for quantitative reconstruction of PET images still remains a challenge in hybrid PET/MRI systems, mainly because cortical bone structures are indistinguishable from proximal air cavities in conventional MR images. Recently, development of short echo-time (STE) MR imaging sequences, has shown promise in differentiating cortical bone from air. However, on STE-MR images, the bone appears with discontinuous boundaries. Therefore, segmentation techniques based on intensity classification, such as thresholding or fuzzy C-means, fail to homogeneously delineate bone boundaries, especially in the presence of intrinsic noise and intensity inhomogeneity. Consequently, they cannot be fully automatized, must be fine-tuned on the case-by-case basis, and require additional morphological operations for segmentation refinement. To overcome the mentioned problems, in this study, we introduce a new fully automatic and reproducible STE-MR segmentation approach exploiting level-set in a clustering-based intensity inhomogeneity correction framework to reliably delineate bone from soft tissue and air.

MR images were acquired on a clinical 1.5T MRI System, MAGNETOM Avanto, using a FLASH 3D pulse sequence with *TE*=1.1*ms*, *TR*=12*ms*, *flip angle*=18°, *voxel size*=1.2×1.2×2*mm*^3^.

For segmentation of the STE-MR images into three regions, consisting of bone, air and soft tissue, a region-based level-set segmentation algorithm was applied. In this technique, k-means clustering is applied to estimate the intensity properties of each region for bias field correction simultaneously with the level-set segmentation. This algorithm incorporates both intensity and spatial information to define continuous boundaries.

The quantitative assessment outcomes of the segmentation performance yielded an average of 89%, 82%, 91%, and 73% for the accuracy, sensitivity, specificity and dice scores in bone segmentation, respectively.

The results suggest that the proposed fully automatic segmentation approach can reliably discriminate bony structures from the neighboring air and soft tissue in STE-MR images, which is suitable for generating accurate *μ*-maps in clinical PET/MR applications.

